# A CT-based radiomics nomogram for classification of intraparenchymal hyperdense areas in patients with acute ischemic stroke following mechanical thrombectomy treatment

**DOI:** 10.3389/fnins.2022.1061745

**Published:** 2023-01-10

**Authors:** Yuan Ma, Jia Wang, Hongying Zhang, Hongmei Li, Fu'an Wang, Penghua Lv, Jing Ye

**Affiliations:** ^1^Department of Interventional Radiology, Northern Jiangsu People's Hospital, Yangzhou, China; ^2^Clinical Medical College, Yangzhou University, Yangzhou, China; ^3^Department of Radiology, Northern Jiangsu People's Hospital, Yangzhou, China

**Keywords:** nomogram, non-contrast-enhanced CT, intraparenchymal hyperdense areas, acute ischemic stroke, mechanical thrombectomy

## Abstract

**Objectives:**

To develop and validate a radiomic-based model for differentiating hemorrhage from iodinated contrast extravasation of intraparenchymal hyperdense areas (HDA) following mechanical thrombectomy treatment in acute ischemic stroke.

**Methods:**

A total of 100 and four patients with intraparenchymal HDA on initial post-operative CT were included in this study. The patients who met criteria were divided into a primary and a validation cohort. A training cohort was constructed using Synthetic Minority Oversampling Technique on the primary cohort to achieve group balance. Thereafter, a radiomics score was calculated and the radiomic model was constructed. Clinical factors were assessed to build clinical model. Combined with the Rad-score and independent clinical factors, a combined model was constructed. Different models were assessed using the area under the receiver operator characteristic curves. The combined model was visualized as nomogram, and assessed with calibration and clinical usefulness.

**Results:**

Cardiogenic diseases, intraoperative tirofiban administration and preoperative national institute of health stroke scale were selected as independent predictors to construct the clinical model with area under curve (AUC) of 0.756 and 0.693 in the training and validation cohort, respectively. Our data demonstrated that the radiomic model showed good discrimination in the training (AUC, 0.955) and validation cohort (AUC, 0.869). The combined nomogram model showed optimal discrimination in the training (AUC, 0.972) and validation cohort (AUC, 0.926). Decision curve analysis demonstrated the combined model had a higher overall net benefit in differentiating hemorrhage from iodinated contrast extravasation in terms of clinical usefulness.

**Conclusions:**

The nomogram shows favorable efficacy for differentiating hemorrhage from iodinated contrast extravasation, which might provide an individualized tool for precision therapy.

## Introduction

Achieving rapid recanalization and reperfusion has been associated with improved clinical outcomes and reduced complications in patients with acute ischemic stroke (AIS) (Linfante et al., 2016). Mechanical thrombectomy (MTB) is a standard treatment option for AIS secondary to large vessel occlusion and has been demonstrated to significantly improve functional outcomes (Berkhemer et al., 2015; Powers et al., 2019). The hyperdense areas (HDA) on postprocedural non-contrast-enhanced CT(NECT) following MTB are common occurrences, which might be secondary to hemorrhage or contrast extravasation (Parrilla et al., 2012; Phan et al., 2012; Lummel et al., 2014). Previous data showed that the presence of HDA suggested a possible association with clinical prognosis (Nakano et al., 2001). However, it can be difficult to differentiate HDA resulting from iodinated contrast vs. that arising from intracranial hemorrhage. Intracerebral hemorrhage is the most feared complication post-MTB with an incidence of 10.9 to 15% (Yoon et al., 2004). Hemorrhage may continue to develop, leading to a marked deterioration with a mortality of up to 83% (Yoon et al., 2004). Therefore, the early and accurate identification of composition of HDA lesions can alter clinical management when antithrombotic therapy is being considered. Thus, ability to discriminate hemorrhage from contrast extravasation in these HDA lesions on NECT is crucial and can have significant clinical worth for AIS patients.

Radiomics can facilitate better clinical decision by improving the process of detecting heterogeneous findings without visible abnormalities in medical images through high-throughput quantitative analysis of statistical features (Gillies et al., 2016). Successful applications of radiomics in acute stroke have been reported in prediction of the hematoma expansion (Ma et al., 2019; Xie et al., 2020; Liu et al., 2021; Song et al., 2021), successful recanalization (Qiu et al., 2019; Hofmeister et al., 2020), recurrence (Tang et al., 2022) and functional outcome (Haider et al., 2021; Quan et al., 2021; Wang et al., 2021). The discrimination of hematomas etiologies (Zhang et al., 2019; Nawabi et al., 2020) using radiomics analysis had been reported as well. These studies showed that radiomics analysis is a feasible and powerful method for guiding diagnosis and treatment in acute stroke.

In this study, we hypothesized that quantitative radiomic features extracted from HDA on NECT images may be used to reflect the composition of contrast material and blood contents. We aimed to develop a model which combines both NECT-based radiomics and clinical risk factors for the classification of hemorrhage and iodinated contrast extravasation following MTB treatment.

## Materials and methods

### Patients

Three hundred and ninety-eight consecutive patients with AIS who underwent MTB treatment from January 2018 to February 2022 in our institution were screened for inclusion into this study. This retrospective study was approved by the institutional review board (No. 2022ky058) and the requirement for written informed consent was waived.

**Figure 1:**
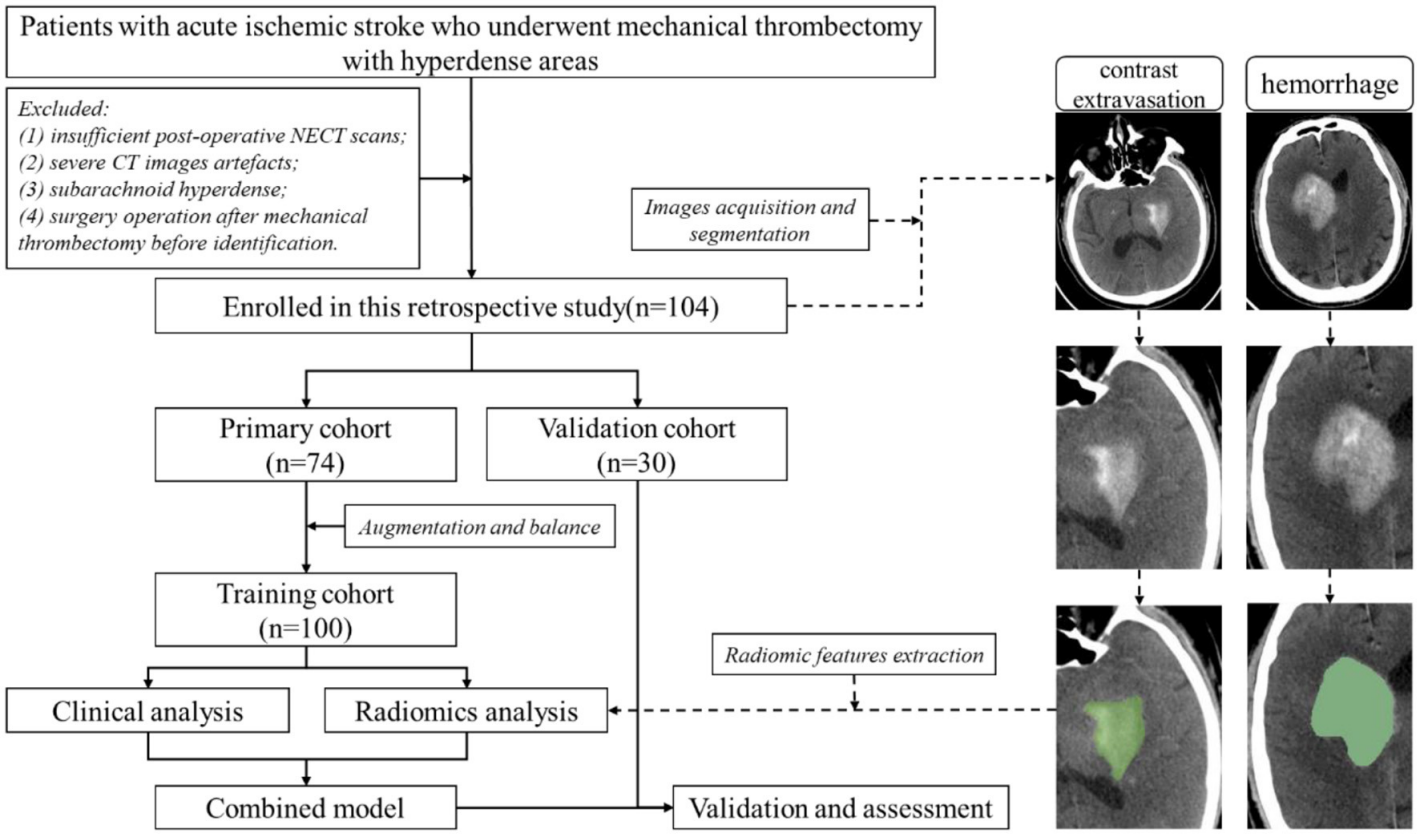
The flow chart of patients' enrollment and image analysis procedure.

The following patients were excluded: (1) without HDA in the initial post-operative NECT scan after MTB within 24 h; (2) severe CT images artifacts; (3) subarachnoid hyperdense; and (4) patients underwent surgery operation after MTB before definitive identification of HDA. The patients' enrollment flow chart was illustrated in Figure 1. Finally, one hundred and four patients with intraparenchymal HDA (mean age, 70.19 years; age range, 35–89 years) were included (Figure 1). The patients were randomly categorized into a primary cohort (*n* = 74) and a validation cohort (*n* = 30) at a ratio of 7:3. Demographic and clinical information of the patients was obtained from medical records.

The presence of HDA was defined emergence of new hyperdensities compared to the surrounding brain tissues exhibited on the initial post-operative NECT scan (< 24 h). Follow-up NECT were scanned consecutively on the next days (>48 h). Contrast extravasation group was classified when hyperdense washout or near-complete cleared within 48 h on NECT scan. Hemorrhage group was classified when hyperdense persisted longer than 48 h (Parrilla et al., 2012; Phan et al., 2012). A dual energy CT(DECT) or MR image was used as the reference standard when available. All images were evaluated by a neuroradiologist and an interventional radiologist with consensus review.

### Image acquisition and analysis

All initial post-operative and follow-up CT images were obtained from three multi-slice CT scanners (SOMATOM Force, Siemens Healthcare; uCT 710, United Imaging Healthcare; LightSpeed VCT 64, GE Medical Healthcare). The scanning was performed using a standard clinical protocols with an axial technique of 5-mm section thickness and reconstruction interval, as well as a scanning energy of 120 kVp tube voltage and an automatic tube current.

### Clinical analysis

The experienced neuroradiologist reviewed the medical records of patients to assess the clinical factors. Univariate analysis was used to compare the differences of clinical factors between the two groups, and a multiple logistic regression analysis was applied to build the clinical model by using the significant risk factors.

### Radiomics analysis

A region of interest (ROI) was manually segmented along the intraparenchymal HDA contour on each initial post-operative NECT image using the 3D-Slicer software (version 4.10.2, www.slicer.org) (Figure 1) by a neuroradiologist with 5 years' experience. Another radiologist with 3 years of experience re-segmented the lesions to evaluate the inter-observer agreement of feature extraction. Both radiologists were blinded to the clinical information and ultimate outcome.

Before feature extraction, raw NECT images were pre-processed to minimize the influence of different scanners. Images were spatially resampled to 1 × 1 × 1 mm, then signal intensity values were discretized to a bin width of 25 with relative intensity rescaling (Hinzpeter et al., 2022). Subsequently, 1316 features were extracted from each ROI by using the 3D Slicer-integrated pyradiomics (http://pyradiomics.readthedocs.io) platform, including seven different categories: shape, first order, gray-level cooccurrence matrix (GLCM), gray-level run length matrix (GLRLM), gray-level size zone matrix (GLSZM), Neighboring Gray Tone Difference Matrix (NGTDM) and gray-level dependence matrix (GLDM) (https://pyradiomics.readthedocs.io/en/latest/index.html). On each feature matrix, additional wavelet filtering and Laplacian of Gaussian filters were applied.

The reproducibility of the radiomics features was analyzed by intraclass correlation coefficient (ICC). An ICC of >0.75 was considered to represent good agreement. To deal with the imbalanced distribution of two groups (54 patients with hemorrhage and 20 patients with contrast extravasation) and avoid model overfitting in the primary cohort, we employed the Synthetic Minority Oversampling Technique (SMOTE). So that the ratio of two groups of HDA patients was improved from 2.7: 1 to 1.5: 1 (60 patients with hemorrhage and 40 patients with contrast extravasation) in the training cohort.

After normalization of the remaining features using z-score standardization, the nonparametric test and least absolute shrinkage selection operation (LASSO) algorithm were applied for dimension reduction and feature elimination. A 10-fold cross-validation was performed during parameter tuning (λ) and valuable feature selection based on the training cohort. In addition, a radiomic score (Rad-score) was calculated using a linear combination of selected features weighted by their respective coefficients, and then radiomic model was constructed.

### Development of combined nomogram model and assessment of different models

A combined model was built by incorporating significant clinical factors as well as Rad-score. To evaluate the calibration and goodness-of-fit of the combined model, the calibration curve and Hosmer–Lemeshow test were assessed. The diagnostic performance of the clinical model, radiomic model and combined model for differentiating hemorrhage from contrast extravasation were evaluated using the receiver operator characteristic (ROC) curves on both the training and validation cohort. The decision curve analysis (DCA) was also performed to calculate the net benefits for a range of threshold probabilities, in order to assess the clinical usefulness of the combined model. The entire feature selection and model fitting process were performed only on the training cohort and evaluated on the validation cohort.

### Statistical analysis

Statistical analysis was performed using SPSS 22.0 software (version 22) and R software (version 3.6.2; www.R-project.org). Continuous variables were presented as mean ± standard deviation or median (interquartile range) as appropriate, while categorical variables were summarized using counts (percentage). The Mann-Whitney *U*-test, independent *t*-test, chi-square test, and Fisher exact test were used as appropriate for univariate analysis.

The area under the ROC curve (AUC), sensitivity, and specificity were then determined using the Youden index. The comparisons of ROCs were accomplished using the DeLong test by Medcalc software (version 15.6.1). In addition, calibration curves along with the Hosmer–Lemeshow test were used to determine the calibration of the combined model. A two paired *p* < 0.05 was considered statistically significant, and a *p* < 0.1 was incorporated into multiple logistic regression analysis.

## Results

### Patient characteristics

Demographic and clinical information of patients in the primary cohort is presented in Table 1. Our data showed that the rate of hemorrhage was 72.97% (54 of 74) and 73.33% (22 of 30) in the primary and validation cohort, respectively. Only cardiogenic diseases, intraoperative tirofiban administration, as well as average and maximum CT value of HDA showed significant differences between patients with hemorrhage and contrast extravasation on primary cohort (*p* < 0.05).

**Table 1:** Clinical factors of the primary cohort.

**Clinical factors**	**Iodinated contrast extravasation (*n* = 20)**	**Hemorrhage (*n* = 54)**	***P*-value**
Age, years	67.00 ± 11.75	69.63 ± 8.41	0.299
Gender, male (%)	12 (60.0)	32 (59.3)	1.00
Hypertension, presence (%)	9 (45.0)	33 (61.1)	0.279
Diabetes mellitus, presence (%)	3 (15.0)	19 (35.2)	0.147
Cardiogenic diseases, presence (%)	4 (20.0)	28 (51.9)	0.017
Preoperative intravascular thrombolysis, Yes (%)	1 (5.0)	15 (27.8)	0.052
Wake up stroke, Yes (%)	4 (20.0)	8 (14.8)	0.722
Smoking, presence (%)	4 (20.0)	14 (25.9)	0.762
Preoperative NIHSS	19.67 ± 8.423	24.70 ± 9.858	0.082
Preoperative ASPECT	6.71 ± 2.469	5.70 ± 2.724	0.192
Times of thrombectomy	2.85 ± 1.537	2.85 ± 1.764	0.993
Onset to recanalization time, minute	372.63 ± 132.501	399.23 ± 104.334	0.380
Intraoperative tirofiban administration, Yes (%)	15 (75.0)	52 (96.3)	0.013
mTICI, 3 (%)	13 (65.0)	25 (46.3)	0.180
Average CT value of HDA, HU	46.20 ± 7.231	52.49 ± 10.730	0.021
Maximum CT value of HDA, HU	69.11 ± 20.61	87.90 ± 34.659	0.030

NIHSS, National institute of Health Stroke Scale; ASPECT, Alberta Stroke program early CT score; mTICI, modified treatment in cerebral ischemia; HDA, hyperdense areas; HU, Housfield.

### Construction of clinical model

In the training cohort, cardiogenic diseases, intraoperative tirofiban administration, preoperative intravascular thrombolysis, preoperative National institute of Health Stroke Scale (NIHSS), the average and maximum CT value of HDA (*p* < 0.1) entered into a multivariable logistic regression analysis. After calculating variance inflation factor (VIF) and tolerance, there was no collinearity observed in these factors (all VIF < 10, tolerance> 0.1). Cardiogenic diseases, intraoperative tirofiban administration and preoperative NIHSS were selected as independent predictors in multivariable logistic analysis (Table 2). The clinical model was constructed based on the independent factors in the training cohort. The models showed AUCs of 0.756 (95% CI 0.660–0.837) and 0.693 (95% CI 0.499–0.848) in the training and validation cohort, respectively (**Figure 4**, Table 2).

**Table 2:** The multiple logistic regression analysis of the clinical factors.

**Clinical factors**	**Coefficient**	***P*-value**	**Odds ratio**	**95% CI of Odds ratio**
Cardiogenic diseases	−1.820	0.014	0.162	0.038–0.688
Intraoperative tirofiban administration	3.048	0.022	21.081	1.541–288.387
Preoperative NIHSS	0.065	0.045	1.067	1.002–1.136
Average CT value	0.047	0.492	1.049	0.916–1.201
Maximum CT value	0.029	0.359	1.029	0.968–1.094
Preoperative intravascular thrombolysis	−0.023	0.976	0.978	0.229–4.171
Constant	−6.221	0.025	0.002	/

CI, Confidence interval.

### Construction and validation of radiomic model

**Figure 2:**
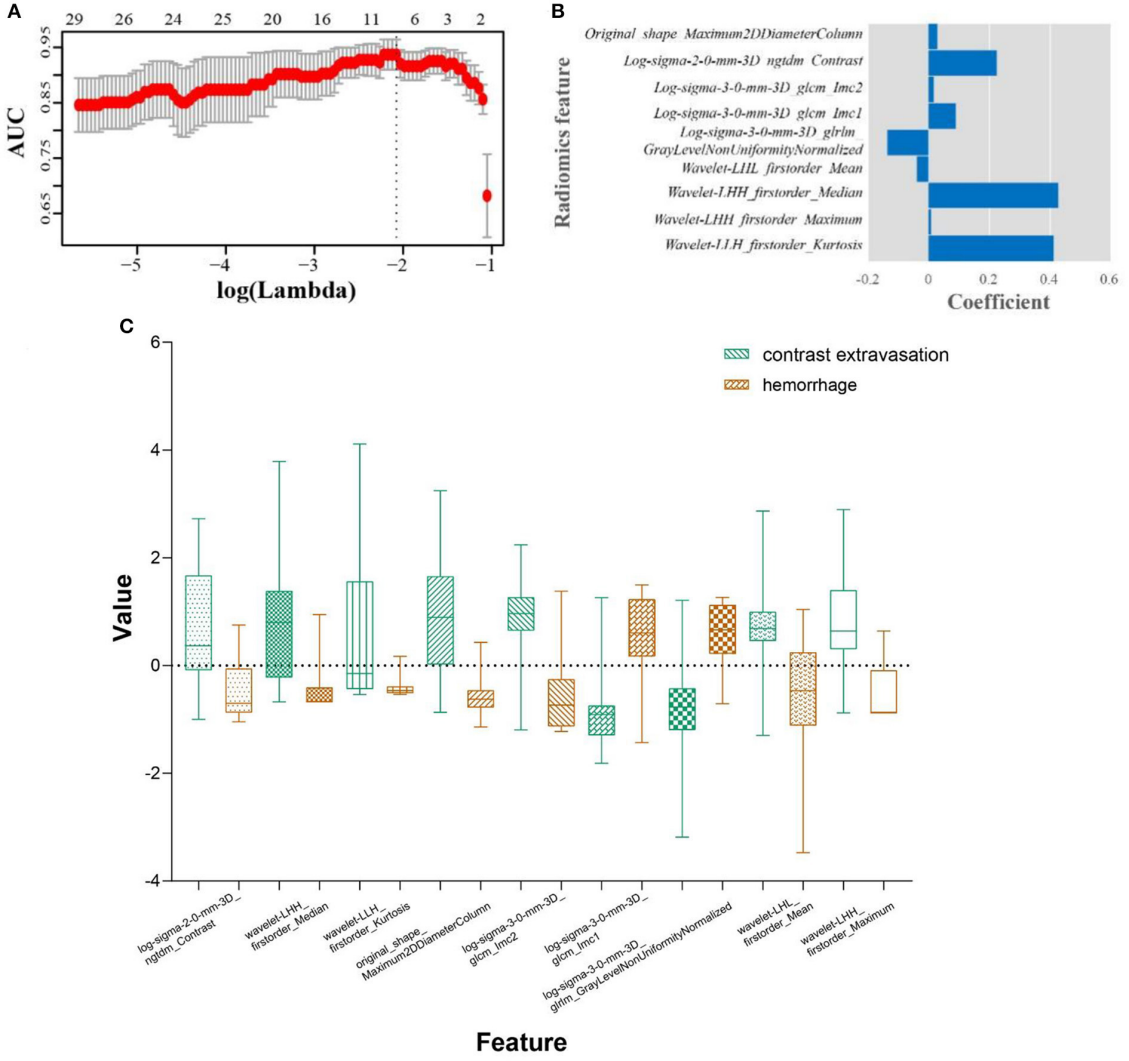
Radiomics feature selection using the least absolute shrinkage and selection operator (LASSO) regression algorithm. **(A)** Tuning parameter (λ) selection in the LASSO used 10-fold cross-validation via minimum criteria. The dotted vertical line was drawn at the optimal values using the minimum criteria. A log (λ) value of −2.079 was opted. **(B)** Boxplots for LASSO coefficient of the selected nine radiomic features. **(C)** Boxplots for the selected features between hemorrhage and contrast extravasation groups.

**Figure 3:**
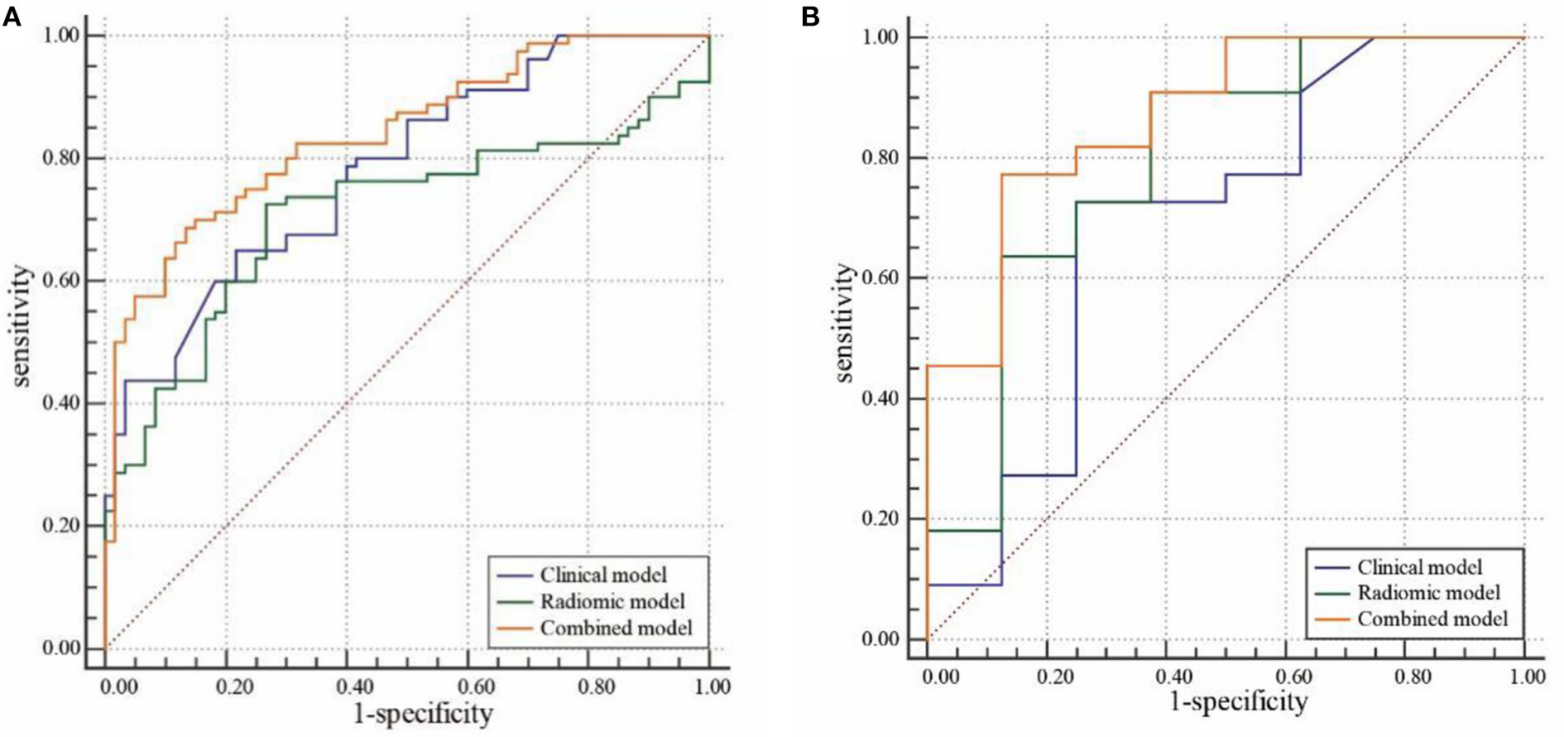
Receiver operating characteristic (ROC) curve analysis of the clinical, radiomic, and combined models in the training cohort **(A)** and validation cohort **(B)**.

Nine robust radiomics features with nonzero coefficients in the LASSO were selected for subsequent modeling with λ = 0.125. The Rad-score was calculated using the following formula: Rad-score = (−0.451) + 0.412 × original_ shape_Maximum2DDiameterColumn + 0.009 × log-sigma-2mm_ngtdm_Contrast + 0.427 × log-sigma-3mm_glcm_Imc2 + (−0.039) × log-sigma-3mm_glcm_Imc1 + (−0.136) × log-sigma-3-0-mm-3D_glrlm_GrayLevelNonUniformityNormalized + 0.089 × wavelet-LHL_firstorder_Mean + 0.016 × wavelet-LHH_firstorder_Median + 0.224 × wavelet-LHH_firstorder_Maximum + 0.028 × wavelet-LLH_firstorder_Kurtosis (Figure 2). The radiomic models showed AUCs of 0.955 (95% CI 0.894–0.986) and 0.869 (95% CI 0.696–0.964) in the training and validation cohort, respectively (Figure 3, Table 2). The detailed definitions and the calculating equations of the selected radiomics features in Supplementary Table 1.

### Construction and validation of combined model

Three clinical factors (cardiogenic diseases, intraoperative tirofiban administration and preoperative NIHSS) and Rad-score were incorporated into the combined model. Our results demonstrated that the combined models showed AUCs of 0.972 (95% CI 0.917–0.994) and 0.926 (95% CI 0.769–0.989) in the training and validation cohort, respectively (Figure 3, Table 3).

**Table 3:** Comparisons of ROC curves of classification models in the training and validation cohort.

**Model**	**AUC**	**95% CI**	**SEN**	**SPE**	**Accuracy**
Training cohort					
Clinical model	0.756	0.660–0.837	0.825	0.600	0.690
Radiomic model	0.955	0.894–0.986	0.900	0.933	0.920
Combined model	0.972	0.917–0.994	0.900	1.000	0.960
Validation cohort					
Clinical model	0.693	0.499–0.848	0.723	0.750	0.743
Radiomic model	0.869	0.696–0.964	0.875	0.773	0.800
Combined model	0.926	0.769–0.989	0.909	0.875	0.884

ROC, receiver operating characteristic; AUC, area under curve; CI, confidence interval; SEN, sensitivity; SPE, specificity.

### Comparison of the classification performance among models

Comparisons of the three models are detailed in Table 3 and Figure 3. In the training cohort, the combined model and radiomic model showed significant greater AUC than the clinical model (AUC: 0.972, 0.955, and 0.756, respectively). In the validation cohort, combined model showed greater AUC than radiomic model and clinical model (AUC: 0.926, 0.869, and 0.693, respectively), but there was no significant difference among models. The comparative analysis of each pair of the three models in training and validation cohorts was showed in Supplementary Tables 2, 3.

### Assessment of models

**Figure 4:**
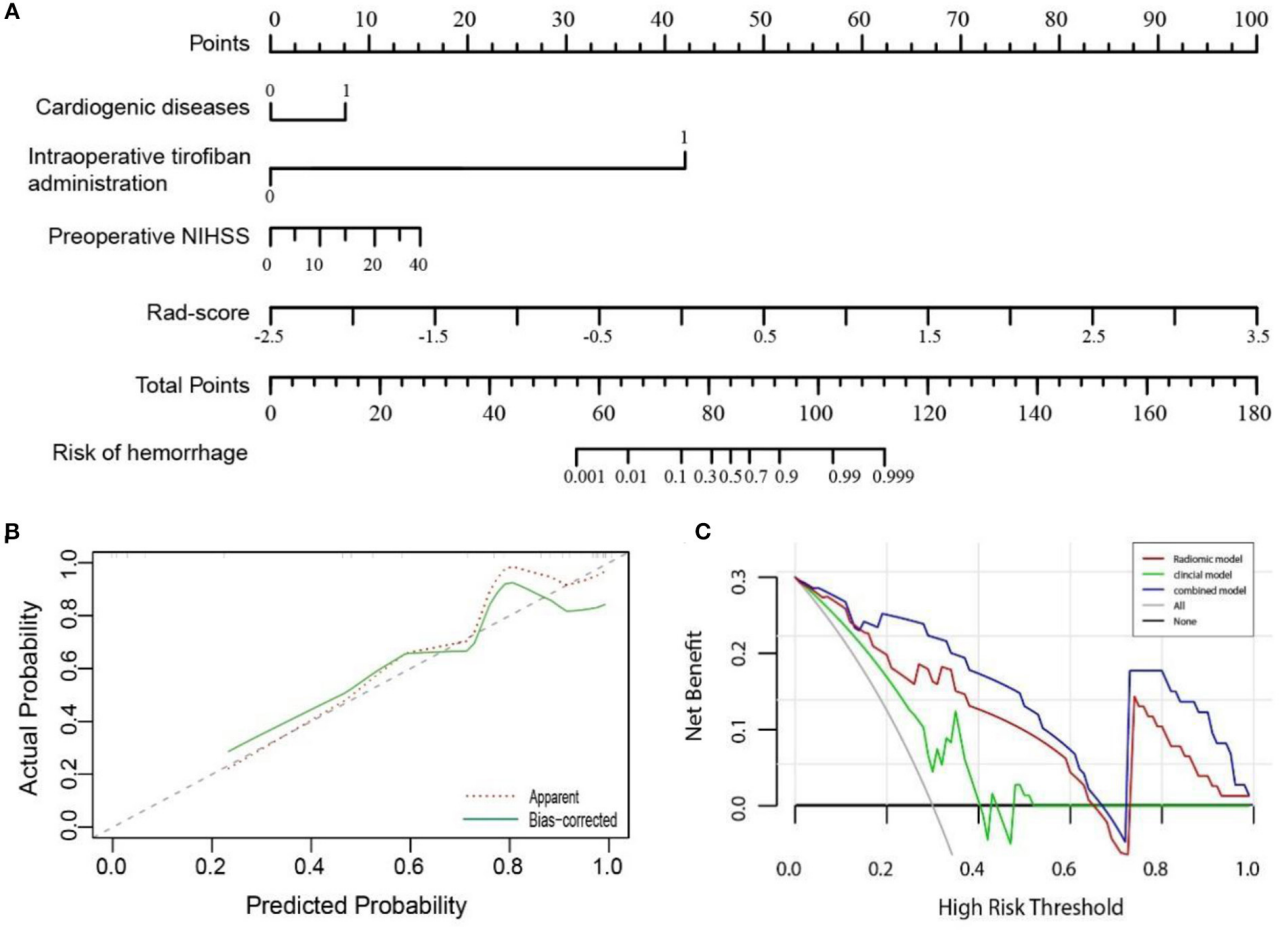
**(A)** A nomogram showing the probability of hemorrhage in hyperdense areas was developed based on the combined model, incorporating into four variables: cardiogenic diseases, intraoperative tirofiban administration, preoperative NIHSS, and Rad-score. **(B)** Calibration curve of the nomogram in the validation cohort. The dashed line was a reference line indicating an optimal nomogram. **(C)** Decision curve analysis for three models in the validation cohort. The y-axis indicates the net benefit; x-axis indicates threshold probability. The blue line, red line, and green line represent net benefit of the combined, the radiomic, and the clinical model, respectively. The combined model had the highest net benefit compared with the other two models across the full range of threshold probabilities at which a patient would be diagnosed as hemorrhage.

A nomogram was performed to visualize the combined model (Figure 4A) for classifying hemorrhage in HDA. The nomogram showed that the Rad-score dominates the scoring system compared with the clinical risk factors, which indicates the significant role of Rad-score in the classification model. The plotted calibration curve showed that the estimative classification of HDA was consistent with the actual observation in the validation cohort (Figure 4B). Similarly, the Hosmer–Lemeshow test showed well calibration on validation cohort (*p* = 0.627).

In addition, as demonstrated in the DCA curve, the combined model had a higher overall net benefit than the clinical or radiomic model across the range of reasonable threshold probabilities in classifying hemorrhage and contrast extravasation in the HDA on the validation cohort (Figure 4C).

## Discussion

To our knowledge, the present study is the first to compare the capability for differentiating hemorrhage from contrast extravasation between radiomic and clinical features in patients following MTB treatment suffering from AIS. Our results showed that radiomic model outperformed clinical model, whereas the model combined of Rad-score and clinical risk factors could improve radiomic model's performance. The derived nomogram can differentiate the presence of hemorrhage in HDA with good discrimination and calibration. In case of emergency where dual-energy CT or MRI technique unavailable, this tool may provide an individualized approach for classifying early hemorrhage.

Although previous studies have analyzed various clinical risk factors associated with higher rates of intracerebral hemorrhage after recanalization, some findings remain controversial (Mokin et al., 2012). In our study, cardiogenic diseases, intraoperative tirofiban administration and preoperative NIHSS were selected as independent predictors for hemorrhage. Tirofiban can competitively inhibit fibrinogen binding to glycoprotein IIb/IIIa receptor, which prevents the platelet aggregation. Previous studies have reported varied results regarding the safety of rescue tirofiban during MTB. Kellert et al. concluded that tirofiban was associated with a higher risk of fatal hemorrhage and poorer prognosis (Kellert et al., 2013), which is consistent with our findings. Current histopathologic studies indicated that the structural composition, histological and biochemical of the clot play a significant role on treatment outcome (Marder et al., 2006; Sporns et al., 2017). Compared to non-cardioembolic thrombi, cardioembolic thrombi has a higher stiffness and resistance to thrombectomy due to a higher proportion of platelets within fibrin-rich areas. Consequently, the characteristic composition of cardioembolic clot aggravate the breakdown of the blood-brain barrier, thereby promoting the extravasation of cellular components from the vessels and ultimately leading to hematoma formation. The correlation of admission NIHSS scores and development of hemorrhage had been confirmed, which higher admission NIHSS scores indicating greater risk of hemorrhage (Tanne et al., 2002).

CT is the first-line imaging modality in evaluating intracranial condition after MTB in clinical practice. The presence of iodinated contrast can be defined by NECT scans, if the attenuation markedly exceeds that expected for hemorrhage (CT values >120 HU). However, hemorrhage cannot be excluded in this situation when iodinated contrast mixed. Definite identification of visualized HDA requires frequent imaging for demonstrating eventual washout which results in increased radiation exposure and expense (Nakano et al., 2001). More importantly, NECT has the shortcoming of a higher false positive rate, as the persist of gadolinium can make the HDA appear to be a hemorrhage when it is truly iodinated contrast extravasation (Gierada and Bae, 1999). As the gold standard for differentiating contrast extravasation vs. iatrogenic hemorrhage, DECT has a high sensitivity and specificity of more than 90% (Phan et al., 2012). However, DECT is limited by potential increase in radiation doses, more expensive, and unavailable in certain hospitals compared to conventional CT (Yedavalli and Sammet, 2017).

The great potential of radiomics analysis for hemorrhagic heterogeneity has been demonstrated by many studies (Zhang et al., 2019; Nawabi et al., 2020). To date, only one study applied CT-based radiomics in differentiating intracranial contrast extravasation from hemorrhage after MTB (Chen et al., 2022). Chen et al. constructed radiomic signature based on initial NECT with AUCs of 0.848 and 0.826 in the training and validation cohort. However, clinical risk factors were not included in this study. Thus, it remains unknown whether radiomic signature are superior to clinical risk factors, and if additional benefit could be yield from the integration of clinical risk factors and radiomic signature. Our results showed that radiomic model outperforms clinical model, whereas the combined model could improve radiomic- or clinical-only model's differential power and yield additional accuracy for HDA classification.

To construct the radiomic model, nine potential radiomic features related to HDA were strictly selected from 1,316 candidate features. Our analysis demonstrated that the log-sigma-2-0-mm-3D_ngtdm_Contrast, wavelet-LHH_firstorder_Median and wavelet-LLH_firstorder_Kurtosis are the features with the highest coefficients. Specifically, contrast is a measure implying the spatial intensity change, and larger range of changes and differences means higher contrast (Amadasun and King, 1989). Kurtosis measures the distribution of intensity values in the image. A higher kurtosis implies that the distribution of signal intensity values tends to the tail(s) rather than the mean, while a lower kurtosis implies the reverse (Zhou et al., 2019). The average values of contrast and kurtosis of hemorrhage group are more than that of contrast extravasation group, which indicating the heterogeneity of hemorrhage was greater than contrast extravasation.

Our study had certain limitations. Firstly, the nature of study is retrospective, single-center, and relatively small samples, which could have led to selection bias and overestimate diagnostic accuracy; thus, a prospective, multi-center, study with external validation in the future is promising. Secondly, manual segmentation is time-consuming and complicated, especially for the lesions with vague boundary. Further study should focus on the advancement of the automatic segmentation technology with satisfactory reliability and reproducibility. Thirdly, the sample size is small and there is an imbalance between the two groups. Although SMOTE was used to achieve group balance, it still exists the possibility of overfitting in the model. Finally, only intraparenchymal HDA were included in this study, subarachnoid hyperdense should be further analyzed.

In conclusion, our study developed a combined nomogram model that showed more favorable differential efficacy in distinguishing hemorrhage from contrast extravasation in HDA following MTB treatment compared to the clinical- or radiomic-only model. The nomogram may provide an individualized tool to supplement the conventional imaging modalities for selecting more patients with hemorrhage that are most likely to benefit from treatment.

## Data availability statement

The raw data supporting the conclusions of this article will be made available by the authors, without undue reservation.

## Ethics statement

The studies involving human participants were reviewed and approved by Northern Jiangsu People's Hospital. Written informed consent for participation was not required for this study in accordance with the national legislation and the institutional requirements.

## Author contributions

PL and JY: guarantor of the article. YM and JW: conception, design, collection, and assembly of data. HZ, HL, and FW: data analysis and interpretation. All authors contributed to the article and approved the submitted version.
