# Supplementary material for: A CT-based radiomics nomogram for classification of intraparenchymal hyperdense areas in patients with acute ischemic stroke following mechanical thrombectomy treatment
Source: Front Neurosci. 2023 Jan 10;16:1061745. doi: 10.3389/fnins.2022.1061745 (PMC9871784; doi:10.3389/fnins.2022.1061745)
Supplement: Supplementary file 1 [file Data_Sheet_1.PDF]

Supplementary Table 1. The detailed definitions and the calculating equations of the selected radiomics features

Image type	Feature Class	Feature name	Definition	Equation
original	Shape	Maximum 2DDiameterColumn	the largest pairwise Euclidean distance between tumor surface mesh vertices in the row-slice (usually the coronal) plane.	/
log-sigma-2mm	NGTDM	Contrast	Contrast is a measure of the spatial intensity change, but is also dependent on the overall gray level dynamic range. Contrast is high when both the dynamic range and the spatial change rate are high, i.e. an image with a large range of gray levels, with large changes between voxels and their neighbourhood.	$\left(\frac{1}{N_{g,p}\left(N_{g,p}-1\right)}\sum_{i=1}^{Ng}\sum_{j=1}^{Ng}p_ip_j\cdot(\mathrm{i}-j)^2\right)\left({}^{1}/_{N_{v,p}}\sum_{1=1}^{N_g}s_i\right)$
log-sigma-3mm	GLCM	Imc2	IMC2 also assesses the correlation between the probability distributions of i and j (quantifying the complexity of the texture). Of interest is to note that HXY1=HXY2 and that HXY2−HXY≥0 represents the mutual information of the 2 distributions. Therefore, the range of IMC2 = [0, 1), with 0 representing the case of 2 independent distributions (no mutual information) and the maximum value representing the case of 2 fully dependent and uniform distributions (maximal mutual information, equal to $\log_2(N_g)$).	$\sqrt{1-\mathrm{e}^{-2(HXY_2-HXY)}}$
log-sigma-3mm	GLCM	Imc1	IMC1 assesses the correlation between the probability distributions of i and j (quantifying the complexity of the texture)	$\frac{HXY-HXY_1}{max\{HX,HY\}}$
log-sigma-3mm	GLRLM	GrayLevelNonUniformityNor	GLNN measures the similarity of gray-level intensity values in the image, where a lower GLNN	$\frac{\Sigma_{\mathrm{i}=1}^{Ng}\left(\Sigma_{j=1}^{Nr}p(\mathrm{i},j\ /\ \theta)\right)^2}{N_r(\theta)^2}$

		malized	value correlates with a greater similarity in intensity values. This is the normalized version of the GLN formula.	
wavelet-LHL	Firstorder	Mean	The average gray level intensity within the ROI.	$^{1}/_{N_P}\sum_{y\geq 1}^{N_P} x\,(\mathrm{i})$
wavelet-LHH	Firstorder	Median	The median gray level intensity within the ROI.	median(X)
wavelet-LHH	Firstorder	Maximum	The maximum gray level intensity within the ROI.	max(X)
wavelet-LLH	Firstorder	Kurtosis	Kurtosis is a measure of the 'peakedness' of the distribution of values in the image ROI. A higher kurtosis implies that the mass of the distribution is concentrated towards the tail(s) rather than towards the mean. A lower kurtosis implies the reverse: that the mass of the distribution is concentrated towards a spike near the Mean value.	$\frac{\frac{1}{Np}\Sigma_{\mathrm{i}=1}^{Np}(x(\mathrm{i})-\overline{\chi})^4}{\left(\frac{1}{Np}\Sigma_{\mathrm{i}=1}^{Np}\left(\chi_{(i)}-\overline{\chi}\right)^2\right)^2}$

NGTDM: Neighbouring Gray Tone Difference Matrix; GLCM: Gray Level Co-occurrence Matrix; GLRLM: Gray Level Run Length Matrix.

Supplementary Table 2. The P-value of comparative analysis of each pair of the three models

	Clinical model	Radiomic model	Combined model
Clinical model	1	0.324	0.107
Radiomic model	<0.001	1	0.202
Combined model	<0.001	0.105	1

The green table cells represent the training cohort, while the red table cells represent the validation cohort.

Supplementary Table 3. The z-statistic of comparative analysis of each pair of the three models

	Clinical model	Radiomic model	Combined model
Clinical model	1	0.987	0.613
Radiomic model	3.745	1	1.275
Combined model	4.252	1.621	1

The green table cells represent the training cohort, while the red table cells represent the validation cohort.
